# Effects of screen time and playing outside on anthropometric measures in preschool aged children

**DOI:** 10.1371/journal.pone.0229708

**Published:** 2020-03-02

**Authors:** Phillipp Schwarzfischer, Dariusz Gruszfeld, Piotr Socha, Veronica Luque, Ricardo Closa-Monasterolo, Déborah Rousseaux, Melissa Moretti, Alice ReDionigi, Elvira Verduci, Berthold Koletzko, Veit Grote

**Affiliations:** 1 Division of Metabolic and Nutritional Medicine, Department of Pediatrics, Dr. von Hauner Children’s Hospital, University Hospital, LMU, Munich, Germany; 2 Neonatal Intensive Care Unit, Children’s Memorial Health Institute, Warsaw, Poland; 3 Department of Gastroenterology, Children’s Memorial Health Institute, Warsaw, Poland; 4 Paediatrics Research Unit, Universitat Rovira i Virgili, IISPV, Reus, Spain; 5 CHC St. Vincent, Liège-Rocourt, Belgium; 6 Department of Paediatrics, San Paolo Hospital, University of Milan, Milan, Italy; Vanderbilt University Medical Center, UNITED STATES

## Abstract

**Objective:**

In view of the current obesity epidemic, studies focusing on the interplay of playing outside (PO), screen time (ST) and anthropometric measures in preschool age are necessary to guide evidence-based public health planning. We therefore investigated the relationship between average time spent PO and ST from the ages 3 to 6 years and anthropometric measures at 6 years of age.

**Methods:**

PO and ST of 526 children of the European Childhood Obesity Project (CHOP) were annually assessed by questionnaire from 3 until 6 years of age. Body weight, waist circumference and height were measured at 3 and 6 years of age to calculate Body-Mass-Index z-Scores (zBMI) and waist-to-height ratio (WTH). Linear, logistic and quantile regressions were used to test whether average time spent PO and ST in the 4 year period had an effect on anthropometric measures at age 6 years.

**Results:**

Longer daily ST was associated with a higher zBMI (P = 0.002) and WTH (P = 0.001) at 6 years of age. No significant associations were found for time spent PO. Each additional hour of average ST during the 4 year period resulted in a 66% higher risk of having a zBMI score over 1 (P < 0.001) and almost twice the risk (94% higher risk) of having an zBMI score over 2 (P < 0.001) at 6 years.

**Conclusions:**

Excessive ST during preschool age is a risk factor for increased zBMI at 6 years, regardless of time spent PO. Reducing high levels of ST during preschool age, for e.g. at least 1h per week, could help preventing childhood obesity.

## Introduction

Over the last decades childhood obesity turned out to be one of the major public health concerns, as long-term consequences on health become more obvious [1]. Changes in body size from 2 to 6 years correlate strongly with adult obesity, which makes this period a prime target for prevention strategies focusing on lifestyle of children [2]. Data suggest that the decline in preschool children’s time spent playing outside (PO) combined with increased screen time (ST) during preschool age could be a modifiable risk factor for childhood obesity, but more evidence is needed [3].

Children’s lifestyle and how they spent their free time have changed in the last decades, with a steady decline of physical activities like outdoor play in 3- to 12-year-olds [4]. However, an active lifestyle can increase daily energy expenditure and thus might be a viable tool to fight the overweight epidemic in addition to a healthy diet [5]. Studies on physical activity in the form of PO have shown promising results. For example, in a one year follow-up study, time spent PO was associated with lower body mass index (BMI) in 3- and 4-year-old children [6]. Another study in 3 year olds reporting decreased overweight risk in children with higher active outdoor play in a cross-sectional analysis [7]. The evidence from these studies is a good indication for beneficial effects of PO, but is restricted to short-term follow-up or cross-sectional samples. Studies looking at the whole preschool period could give further insight to what extend PO can contribute to the prevention of obesity.

While the amount of time spent PO decreases, media use in children and adolescence becomes more and more common [8], and starts ever earlier in childhood [9, 10]. Various guidelines are in place recommending appropriate duration of daily ST for children in different ages [11, 12]. In a recently published policy statement the focus of the American Academy of Pediatrics lies on media use in preschool children, recommending a limit of 1 hour or less per day of high-quality media (i.e. content, which is pedagogically valuable and developed for children) use for children older than two years of age [13]. These recommendations are based on several studies showing that higher ST in younger ages is a risk factor for obesity and delayed development of the child [14–16]. However, evidence from these studies is mostly based on cross-sectional data.

Although pathways might be clear, there is a distinctive lack of prospective studies in preschool children examining the relationship between PO, ST and anthropometric measures at later ages. In order to make this gap smaller we aim to test whether time spent PO or ST from 3 to 6 years of age is associated with anthropometric measures at 6 years of age.

## Methods

### Study subjects and design

The study population used is a subset of the “European Childhood Obesity Project” trial, which was designed as a double-blind randomized control trial registered at clinicaltrials.gov as NCT00338689; URL: clinicaltrials.gov/ct2/show/NCT00338689. Details and the primary outcome are published elsewhere [17, 18]. In short, the intervention consisted of two types of infant and follow-on formulae, one with higher and one with lower protein content. Besides those two intervention groups the study also included an observational group of children, who were exclusively breastfed for at least the first three months of life. Healthy full term infants who were born between 1st October 2002 and 31st July 2004 were recruited in five countries (Germany, Spain, Italy, Poland, Belgium; a total of 11 study centers) during their first 8 weeks of life. Part of this secondary analysis were all children with objective height, weight and waist circumference measurements at 3 years as baseline and at 6 years as follow-up with additional annual questionnaire data on PO and ST assessed at 3, 4, 5 and 6 years of age.

### Ethics statement

The study was conducted according to the principles expressed in the Declaration of Helsinki. The local ethics committees of each study center approved all study procedures: Belgium (Comitè d’Ethique de L’Hopital Universitaire des Enfants Reine Fabiola; no. CEH 14/02), Germany (Bayerische Landesärztekammer Ethik-Kommission; no. 02070), Italy (Azienda Ospedaliera San Paolo Comitato Etico; no. 14/2002), Poland (Instytut Pomnik–Centrum Zdrowia Dziecka Komitet Etyczny; no 243/KE/2001), and Spain (Comité ético de investigación clinica del Hospital Universitario de Tarragona Joan XXIII). Written informed parental consent was obtained for each infant.

### Anthropometric measurement

Children’s weight, height and waist circumference were measured at study centers at the 3 and 6 years follow-up visit. Standard operating procedures based on the World Health Organization’s Multicenter Growth Reference Study were applied [19]. The same equipment was used in all study centers and study personnel were trained repeatedly during the study to ensure reliable results. All measurements were taken twice, and their means were taken for analysis. BMI was calculated in kg/m^2^, which has been proven to be a good measure for child overweight and obesity [20]. Age- and sex-specific BMI z-scores (zBMI) were computed based on the World Health Organization’s reference population [21]. Waist to height ratio (WTH) was calculated as ratio between waist circumference [cm] and height [cm]. WTH has been shown to be a reliable measure of abdominal adiposity in children [22].

### Activity assessment

At the 3 year, 4 year, 5 year and 6 year follow-up questionnaires were handed out, including four questions asking for a typical weekday or weekend day in the last month. A four items questionnaire was used to assess time PO and ST, which was filled by parents before or during study visits. To asses children’s time PO their parents were asked to recall: “How much time would you say your child spends playing outdoors on a typical weekday?” and “How much time would you say your child spends playing outdoors on a typical weekend day?”. For ST similar questions were used: “

1. How much time would you say your child spends watching TV, playing videogames or using the computer on a typical weekday?”
2. “How much time would you say your child spends watching TV, playing video games or using the computer on a typical weekend day?“.

Answers should be entered in free text field in hours and minutes. Identical questionnaires are used in other studies and validated in similar populations [23]. The mean time spent PO and ST per day was calculated as (hours/weekday × 5 + hours/weekend day × 2)/7.

### Covariates

Additional to gender and study country seven covariates were considered in the analysis of which following covariates were collected at study recruitment: highest education level of mother and father according to International Standard Classification of Education 1997 levels, defined as low (level 0–2), middle (level 3–4) and high (level 5–6) [24], pre-pregnancy BMI, calculated from self-reported height and weight of mothers and dichotomized as BMI above and below 25, smoking status during pregnancy, and the child’s birthweight. At 3 years of age children’s caloric intake was assessed with 3-days weighted food protocols filled in by parents [25]. We defined a “season of measurement” variable (spring [Mar-May], summer [Jun-Aug], autumn [Sept-Nov] and winter [Dec-Feb]) based on the visit date.

### Data management

Data are reported as mean (μ) ± standard deviation (SD) for continuous variables and as number (n) and percentage (%) for factors. Main predictor variables were time spent PO and ST. Implausible values of more than 10 hours of ST per day were excluded (4 data points). Both PO and ST showed to be relatively stable behaviors over the preschool period: Spearman correlation coefficients of successive PO measurements ranged from 0.51 to 0.60; correlation coefficients of successive ST measurements coefficients ranged from 0.60 to 0.64. We combined consecutive measurements of PO and ST into one average PO and one average ST variable, to estimate the average time spent PO and ST over the whole preschool period. Variables PO and ST used in the data analysis were calculated as individual means of children’s PO and ST measurements over the 4 years period. As follow-up of all participants at all time points was not achieved, we defined that only children with at least two measurement points with both PO and ST data at the respective time point were included in the analysis. We defined overweight as a zBMI at 6 years >1, obesity as a zBMI at 6 years >2 and WTH at 6 years > 0.5 as a measure of increased central adipose tissue associated with worsened cardiometabolic risk [20, 26].

### Data analysis

In the primary analysis we looked at effects of average time spent PO and average ST on anthropometric outcomes: Two separate linear regression models were built with zBMI and WTH at 6 years as outcomes and average ST and average time spent PO as main predictors. A third mutually adjusted linear regression model with both PO and ST as predictors was used. All models were adjusted for zBMI or WTH at age 3 years, respectively, intervention group and country. Sex was only added to the WTH model, as zBMI already incorporated sex. Additional covariates (see covariate section) were selected based on highest adjusted R^2^. Children with more measurements points were assumed to have a more precise measure compared to children with less measurement points. Therefore, regression models were weighted according to the number of measurement points of PO and ST (two to four measurement points) available per child. Average PO and ST variables were potentially age biased, as some children only had data from later time points, which had an average higher ST and lower PO.

Secondly, we applied logistic regression to assess the impact of PO and ST on overweight, obesity and increased WTH. Adjustment was identical to linear regression models. Furthermore, to explore whether effect estimates differ by quantiles of zBMI and WTH, quantile regression models for the 10^th^ to 90^th^ percentiles were built in steps of ten. Beta estimates for each quantile were plotted to visualize differences in effect sizes over the zBMI/WTH distribution of the sample.

Additional bias could arise from cross-sectional associations between PO/ST and anthropometrics at the last time point. Therefore sensitivity analysis was conducted with two separate models, one with children, who completed PO and ST measurements with 3 years and 4 years of age. Another model was built with children, who completed PO and ST measurements with 3 years and 4 years and 5 years of age.

Another sensitivity analysis was conducted by employing multiple imputation to avoid bias from missing data. All missing values of outcome and exposure variables from children, who participated at either baseline or follow-up were imputed by a bootstrapping-based algorithm for time series data, resulting in five imputed data sets [27–29]. Estimates of linear and logistic regressions with these five data sets were combined by Rubin’s Rule [30, 31]. Statistical significance was assumed at a maximum error probability of 0.05. Statistical analysis was carried out with R 3.4.3 (The R Foundation for Statistical Computing).

## Results

### Descriptive information

**Fig 1:**
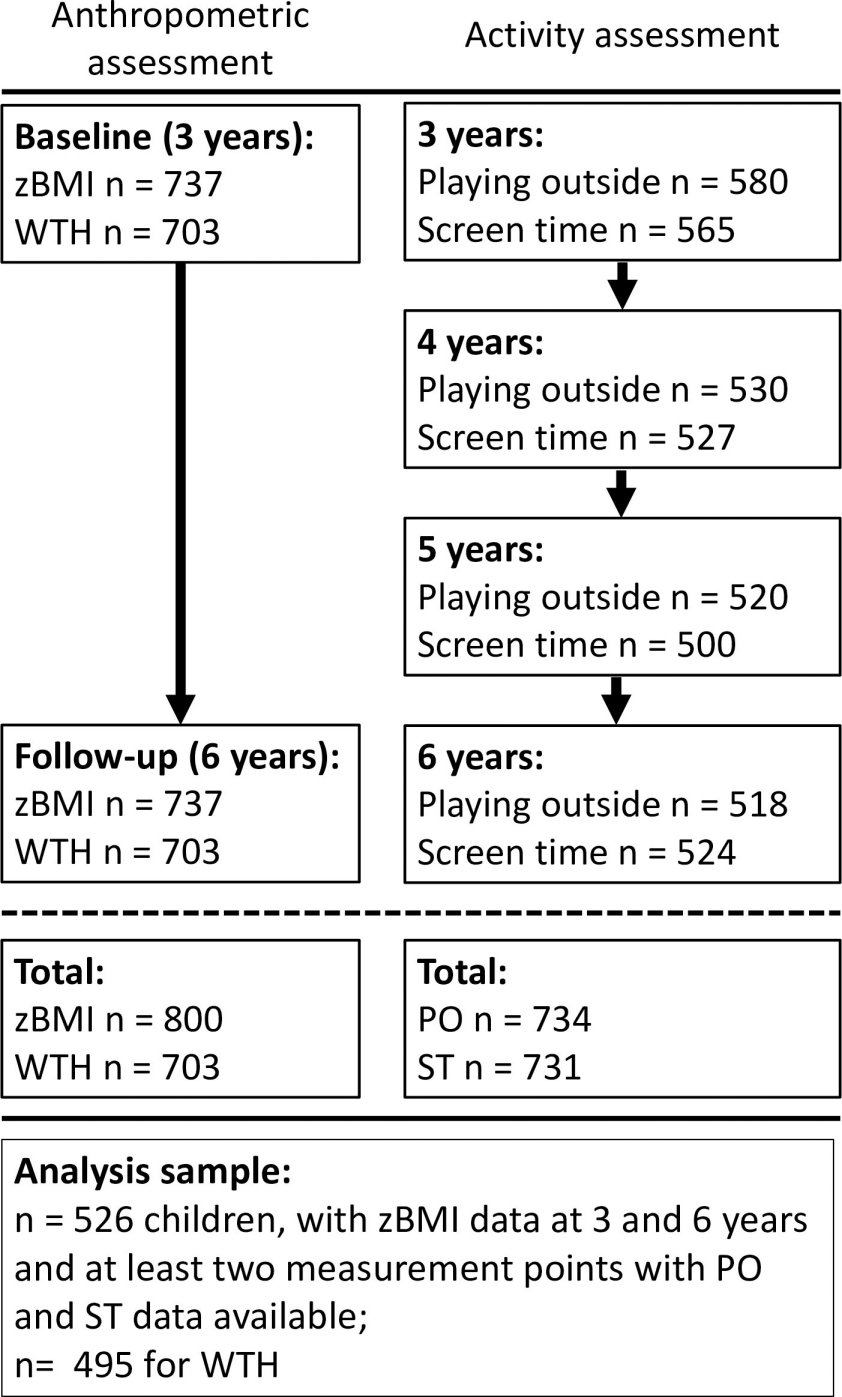
Flow chart of study participation. Abbreviations: zBMI BMI z-scores according to WHO reference population, WTH waist-to-height ratio.

Eight hundred children participated at either baseline or follow-up visit with valid anthropometric data. Of those 800 children at least one PO or ST measurement was available for 734 children, where 731 completed the PO questionnaire and 729 the ST questionnaire at least once. In total 526 children were included in the analysis with zBMI data at 3 and 6 years and at least two measurements of PO and ST points with PO and ST data available; 495 for WTH (Fig 1). Of these children 87 had measurements of PO and ST at two time points, 179 at three time points and 260 at four time points. Table 1 shows the characteristics of children included in the analysis. BMI and zBMI slightly decreased during the 3 years period, but the numbers of children who are classified as overweight or obese increased. Table 2 summarizes the time spent PO and ST at the four measurement points. Average weekly time spent PO showed a slight decrease of 6 min while average weekly ST increased by 21 min, especially on weekend days with a plus of 40 min. Time spent PO and ST was significantly higher on weekend days compared to weekdays at all 4 time points. Sex differences in weekly ST were seen at 3 years of age, were girls had a significantly (P = 0.004) lower average weekly ST (1.0h ± 0.8) than boys (1.3h ± 1.0) (S1 Table). PO differed by sex at 6 years of age (Female mean: 2.37h ± 1.62; Male mean: 2.80h ± 2.13; P = 0.02) (S1 Table). Children from family with a low education level spent the most time PO (Low: 3.27h ± 2.00; Middle: 2.62h ± 1.45; High: 2.41h ± 1.24; P <0.001) and children from high educated families the least amount of time in front of a screen (High: 1.14h ± 0.68; Middle: 1.42h ± 0.77; Low: 1.59h ± 0.87: P <0.001). Country differences were seen in baseline and follow-up zBMI scores, with highest values in Italy (Baseline zBMI: 0.56 ± 0.95; Follow-up: 0.52 ± 1.22). Average ST from 3 to 6 years of age was lowest in Germany (0.57 ± 0.45) and highest in Poland (1.86 ± 0.74) (S2 Table).

**Table 1:** Age, sex, country and anthropometrics at 3 and 6 years of age of 526 study participants with at least 2 measurements of playing outside or screen time at different time points from 3 to 6 years of age.

	3 years	6 years
Sex = Male (%)	245 (46.6)	
Country (%)		
Belgium	90 (17.1)	
Spain	137 (26.0)	
Germany	61 (11.6)	
Italy	159 (30.2)	
Poland	79 (15.0)	
Age (mean (sd))	3.03 (0.06)	6.03 (0.07)
BMI (mean (sd))	15.97 (1.33)	15.94 (1.99)
zBMI (mean (sd))	0.31 (0.98)	0.30 (1.16)
BMI Categories (%)		
normal	502 (95.4)	404 (76.8)
obese	5 (1.0)	36 (6.8)
overweight	17 (3.2)	81 (15.4)
underweight	2 (0.4)	5 (1.0)
Waist-to-height ratio (n = 495)	0.52 (0.03)	0.47 (0.04)

In total data 800 children participated at either baseline or follow-up visits, of whom 526 had both baseline and follow-up BMI measured and completed the annual “playing outside” and “screen time” questionnaire at least twice.

Abbreviations: BMI Body mass index, zBMI BMI z-scores according to WHO reference population

**Table 2:** Time spent playing outside and screen time of children at each time point and the average over all time points.

Age (years)	3	4	5	6	Average
Playing outside (mean h/day (SD)),					
n	458	456	436	461	526
PO weekday	2.48 (1.91)	2.29 (1.71)	2.31 (1.86)	2.33 (2.00)	2.35 (1.42)
PO weekend	3.16 (1.95)	3.15 (1.87)	3.07 (1.98)	3.19 (2.13)	3.15 (1.67)
PO average per week	2.68 (1.76)	2.52 (1.60)	2.52 (1.73)	2.58 (1.89)	2.58 (1.41)
Screen time (mean h/day (SD))					
n	447	452	421	460	526
ST weekday	1.10 (0.94)	1.16 (0.88)	1.31 (0.91)	1.30 (0.89)	1.20 (0.73)
ST weekend	1.31 (1.11)	1.55 (1.14)	1.80 (1.21)	1.99 (1.31)	1.65 (0.95)
ST average per week	1.15 (0.91)	1.27 (0.89)	1.44 (0.91)	1.50 (0.93)	1.33 (0.75)

Abbreviations: n Sample size, SD standard deviation, PO playing outside, ST screen time

### Associations between PO and ST and anthropometric measures

Table 3 shows the associations between average time spent PO and ST between the ages of 3 to 6 years on anthropometrics at 6 years of age. Significant positive association were found between ST and zBMI (P = 0.002) and WTH (P = 0.001). Similar results were seen in a mutually adjusted model, with both PO and ST in the model. The unadjusted base models showed analogous results (S3 Table).

**Table 3:** Associations between average time spent playing outside and in front of a screen from 3 to 6 years of age on body mass index z-score and waist-to-height ratio at 6 years.

	Separate model for playing outside (PO) and screen time (ST)				Mutually adjusted models for PO and ST	
	zBMI	WTH	zBMI	WTH	zBMI	WTH
	ß (95% CI)	ß (95% CI)	ß (95% CI)	ß (95% CI)	ß (95% CI)	ß (95% CI)
PO	-0.002	0			0	0
	(-0.05, 0.04)	(-0.002, 0.002)			(-0.05, 0.05)	(-0.002, 0.002)
ST			0.15*	0.01*	0.15*	0.01*
			**(0.06, 0.25)**	**(0.003, 0.01)**	**(0.06, 0.25)**	**(0.003, 0.01)**
n	508	477	508	477	508	477
R^2^	0.60	0.37	0.60	0.38	0.60	0.38
Adjusted R^2^	0.59	0.36	0.59	0.37	0.60	0.37

All ß coefficients from linear regression models, adjusted for country, intervention type, baseline anthropometrics and maternal pre-pregnancy BMI (categorized in above and below 25).

Abbreviations: PO playing outside, ST screen time, 95% CI 95% confidence interval, zBMI BMI z-scores according to WHO reference population, WTH waist-to-height ratio;

* p < 0.001

Table 4 shows the effects of average time spent PO and ST from 3 to 6 years of age on the risk for overweight or obesity at 6 years of age. One hour of additional ST per day significantly increased the odds of having a zBMI greater than one (Odds Ratio: 1.66, 95% CI: 1.51–1.80, P < 0.001) or two (OR: 1.94, 95% CI: 1.72–2.17, P < 0.001) at 6 years of age. Similar effects were seen when looking at the odds for having a WTH higher than 0.5 at 6 years of age. Effects per additional hour of daily time spent PO showed a risk reducing effect (P < 0.001).

**Table 4:** Effects per hour of average time spent playing outside and in front of a screen from 3 to 6 years of age on the odds of having a zBMI >1,zBMI > 2 or WTH > 0.5 at 6 years.

	zBMI >1	zBMI > 2	WTH > 0.5
	OR (95% CI)	OR (95% CI)	OR (95% CI)
PO	0.85*	1.08	0.99
	**(0.77, 0.91)**	(0.97, 1.19)	(0.92, 1.07)
ST	1.66*	1.94*	1.78*
	**(1.51, 1.80)**	**(1.72, 2.17)**	**(1.61, 1.95)**
n	508	508	477

All odds ratios from logistic regression models, adjusted for country, intervention type, baseline anthropometrics and maternal pre-pregnancy BMI (categorized in above and below 25). Abbreviations: PO playing outside, ST screen time, 95% CI 95% confidence interval, zBMI BMI z-scores according to WHO reference population, WTH waist-to-height ratio;

* p < 0.001

**Fig 2:**
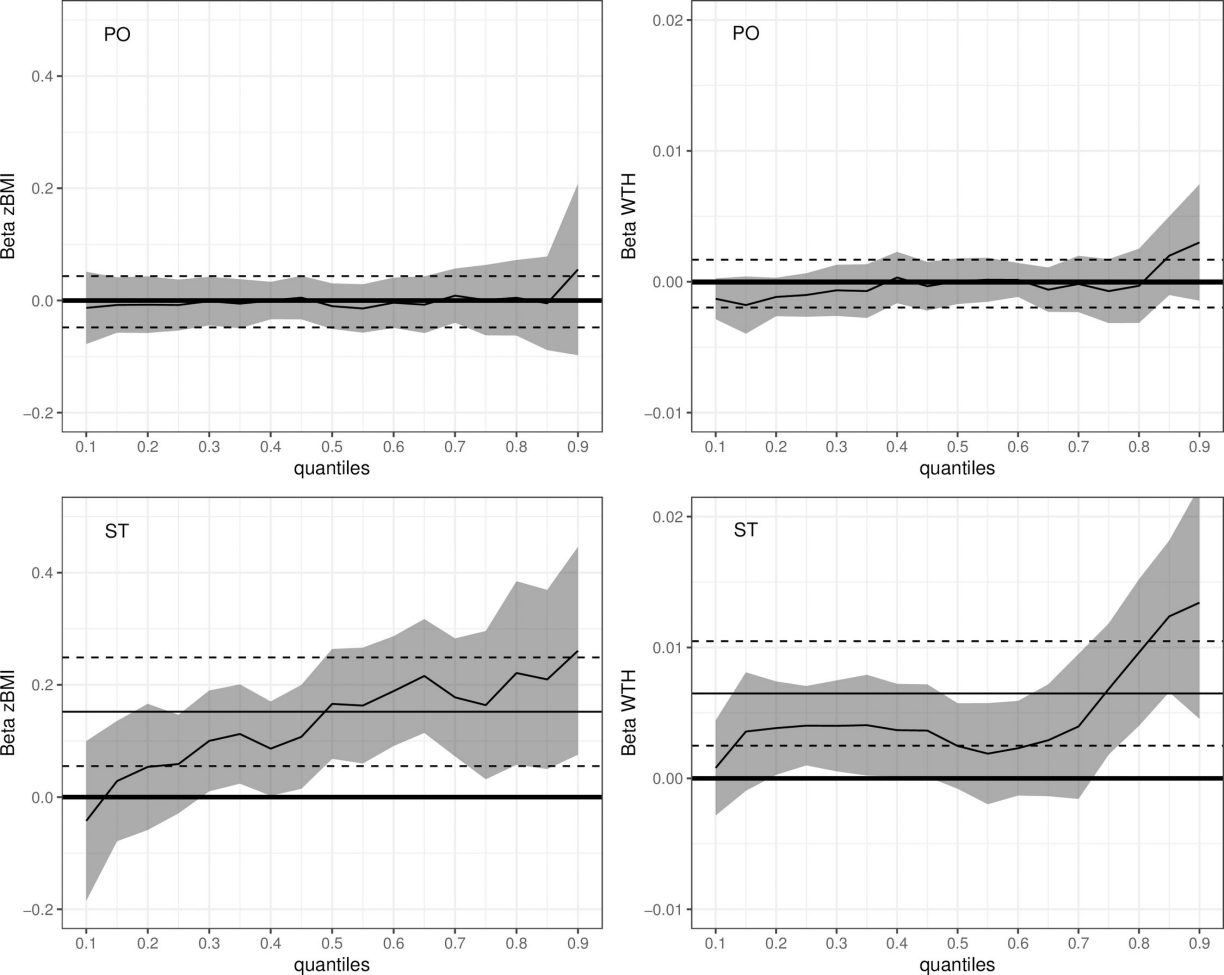
Plotted beta estimates of association between time playing outside (PO) and screen time (ST) between 3 to 6 years with anthropometrics (BMI z-score and waist-to-height ratio, WTH) at 6 years per percentile, calculated by quantile regression. Horizontal lines indicated estimates from linear regression model and 95% CI (dashed lines); grey area indicate 95% CI of each percentile’s estimates. Quantile regression models were adjusted for baseline anthropometrics, sex, country, intervention type, BMI of mother before birth and mutually adjusted for ST and PO.

Fig 2 shows results of the quantile regression models for the 10^th^ to 90^th^ percentile of zBMI and WTH. Effect estimates for PO were similar across all percentiles of the zBMI and WTH distribution. Looking at ST, higher effect estimates were observed for the upper percentiles of zBMI (50^th^ percentile β_ST_ = 0.17, P < 0.001; 90^th^ percentile β_ST_ = 0.26, P = 0.006). For WTH effects start to increase from the 70^th^ percentile upwards (70^th^ percentile β_ST_ = 0.004, P = 0.161; 90^th^ percentile β_ST_ = 0.01, P = 0.003).

Sensitivity analysis using only the average PO and ST of the first three (S4 Table) and two measurement time points (S5 Table) was performed. In short, results did not differ in estimates and significance compared to results from the linear regression model with all available time points. Results of the imputed dataset, which included complete data for 800 children (S6 Table, S7 Table) showed no change of significance. Estimated effects for ST were slightly higher in linear and logistic regression models.

## Discussion

### Principal findings

Our analyses showed that average ST between 3 and 6 years was associated with a higher zBMI and WTH at 6 years, independently of average time spent PO in the same time frame. An hour of additional ST per day resulted in an almost twofold risk of being obese at 6 years of age. Effect of ST differed by zBMI and WHT percentile, where the upper percentiles representing overweight and obese children showed a greater increase of zBMI and WTH than normal weight children. These results emphasize the need for early intervention to focus on reduction of sedentary activities like ST, especially in already overweight children.

### Implications and comparison with other studies

Literature support the results of our study of a positive association between ST and body size, independent of time spent PO. A cross-sectional analysis of 759 preschool children from the GECKO Drenthe cohort found that long ST is related to a higher BMI [32]. In the same study outdoor play was inversely associated with ST, but not with BMI. Another cross-sectional study used data of 7505 5-years-old children to look at the relationship between nutrition, PO and watching television. Results showed that watching television for more than 2h/day was associated with a higher risk of being overweight or obese, whereas PO had no effect on body size [33]. We could not confirm a protective effect of PO against the development of overweight due to increased active energy expenditure, which was seen in other studies [6, 34]. One possible explanation could be, that in younger age outdoor activities are not necessarily different from indoor activities from an energy expenditure perspective, e.g. sitting in the sandbox at the playground and sitting at home playing with toys. Another reason might be that motor skills (like sustained running or jumping) are still in development during early life, meaning smaller children are not as capable of performing high energy expenditure activities as older children. This might explain why effects of PO and physical activity as a whole are more consistent at later ages [34–36].

Our study and concurrent literature confirm that negative effects of ST are already consistent in young children. Kuhl et al. describe ST as a major behavioral correlate of obesity in preschoolers and emphasize the need for intervention [3]. Two other more recent studies found similar results, in cross-sectional designed trials [37, 38]. Based on this consistent evidence interventions should start early, to prevent an establishment of excessive ST in the lifestyle of children which tracks into later life [9, 10]. With the ever faster change of digital media, raising parent’s awareness of risks and opportunities of ST in the early life of their children should be strongly considered by health care professionals and in public health planning [39, 40]. For a recommendation of ST reduction, more practical values like hours per week, can be helpful for parents. A study by Wen et al. in 2 years old children for example found that for every additional hour per week the risk for overweight and obesity increased by 10% [41]. In our sample a similar effect was seen for the risk of having a zBMI > 1 (7% for every hour ST per week, P < 0.001) and zBMI > 2 (10% for every hour ST per week, P < 0.001; S8 Table).

One possible explanation for the negative effects of ST is its relationship to negative dietary habits. Two recent studies found that increased ST is associated with higher energy intake, less consumption of vegetables and fruits and a higher consumption of sugar-sweetened beverages [42–44]. A recent systematic literature review by Hale and Guan additionally found significant associations between ST and reduced sleep duration and increased sleep problems [45]. All these behaviors are consistently related to childhood obesity [46–48], but are also more prominent in already overweight children. This might explain that effects of ST are more pronounced in children above the 50^th^ percentile of zBMI.

### Strengths and weaknesses

A definite strength of our study is the longitudinal and multicenter design. This makes it possible to give a statement about the direction of effects in a rather large population group. Especially compared to cross-sectional studies, which often rely on rather locally restricted samples. The results of our study are based on a European birth cohort, which makes results generalizable for children from these five European countries. However, the population might not be fully representative as children were mainly from urban areas and about two thirds of them took part in the intervention during the first year of life [18]. Additionally, some methodological factors limit generalizability to some extent. First of all, measurements of PO and ST were based on self-reported data from parents. Although anonymity was assured and answers were checked for plausibility, parents might have given socially desirable answers or misunderstood the questions. Height and weight of the children were, however, measured by trained health care professionals during well-child visits. Another limitation is the measurement of PO as approximation for physical activity. Subjective measurements of time spent PO with questionnaires are an easy method to implement, but objective measurements with accelerometer are more precise [49]. While these subjective measurements of PO and ST lack precision, their practical implication is more straightforward. Time spent playing outside and screen time are measures, which are easy to understand for parents and public health actors. Therefore, the times can be easier influenced and changed by e.g. actively going outside with the child or restricting ST by rules. Levels of habitual physical activity, as measured by accelerometers, are more difficult to grasp and hard to change as intervention studies have shown [50, 51].

## Conclusion

Results of this study have shown that ST is associated with an increase in zBMI and WTH over the course of 4 years. Each additional hour of ST doubled the risk of becoming overweight or obese at the age of 6 years, independent of baseline weight status and time spent PO. Effects of ST were more pronounced in higher percentiles of zBMI and WTH. PO had little effect on the early anthropometric development of children. Reducing media use in preschool and encouraging children to a more active lifestyle, could help fighting childhood obesity.

## Supporting information

S1 Table Time spent playing outside and screen time of children at each time point, stratified by sex. (DOCX)

S2 Table Body mass index z-scores, waist to height ratio at baseline (3 years) and follow-up (6 years) and average time spent playing outside and in front of a screen over the time period, stratified by country. (DOCX)

S3 Table Associations between average time spent playing outside and in front of a screen from 3 to 6 years of age on body mass index z-score and waist-to-height ratio at 6 years; base model. (DOCX)

S4 Table Associations between average time spent playing outside and in front of a screen from 3 to 5 years of age on body mass index z-score and waist-to-height ratio at 6 years. (DOCX)

S5 Table Associations between average time spent playing outside and in front of a screen from 3 to 4 years of age on body mass index z-score and waist-to-height ratio at 6 years. (DOCX)

S6 Table Associations between average time spent playing outside and in front of a screen from 3 to 6 years of age on body mass index z-score and waist-to-height ratio at 6 years; Imputed dataset. (DOCX)

S7 Table Effects of average time spent playing outside and in front of a screen from 3 to 6 years of age on the odds ratio of having a zBMI >1,zBMI > 2 or WTH > 0.5 at 6 years; Imputed dataset. (DOCX)

S8 Table Effects per hour of average time spent playing outside and in front of a screen per week from 3 to 6 years of age on the odds of having a zBMI >1,zBMI > 2 or WTH > 0.5 at 6 years. (DOCX)

## Abbreviations

95% CI: 95% Confidence Interval
PO: Playing outside
SD: Standard deviation
ST: Screen time
WTH: Waist-to-height ratio
zBMI: Body mass index z-score
