# Supplementary material for: Effects of screen time and playing outside on anthropometric measures in preschool aged children
Source: PLoS One. 2020 Mar 2;15(3):e0229708. doi: 10.1371/journal.pone.0229708 (PMC7051070; doi:10.1371/journal.pone.0229708)
Supplement: S1 Table — (DOCX) [file pone.0229708.s001.docx]

**S1 Table. Time spent playing outside and screen time of children at each time point, stratified by sex**

| Age (years) | 3 | | 4 | | 5 | | 6 | |
| --- | --- | --- | --- | --- | --- | --- | --- | --- |
| Playing outside (mean h/day (SD)), | | | | | | | | |
|  | Female | Male | Female | Male | Female | Male | Female | Male |
| n | 245 | 213 | 242 | 214 | 237 | 199 | 242 | 219 |
| PO weekday | 2.40 (1.80) | 2.57 (2.04) | 2.21 (1.59) | 2.37 (1.83) | 2.16 (1.57) | 2.48 (2.15) | 2.15 (1.70)* | 2.54 (2.28)* |
| PO weekend | 3.04 (1.88) | 3.30 (2.02) | 3.13 (1.81) | 3.16 (1.94) | 3.01 (1.83) | 3.14 (2.14) | 2.96 (1.97)* | 3.43 (2.27)* |
| PO average per week | 2.59 (1.66) | 2.78 (1.86) | 2.47 (1.49) | 2.58 (1.71) | 2.41 (1.49) | 2.66 (1.96) | 2.37 (1.62)* | 2.80 (2.13)* |
| Screen time (mean h/day (SD)) | | | | | | | | |
|  | Female | Male | Female | Male | Female | Male | Female | Male |
| n | 241 | 206 | 240 | 212 | 233 | 188 | 240 | 220 |
| ST weekday | 0.97 (0.83)** | 1.24 (1.03)** | 1.12 (0.90) | 1.20 (0.85) | 1.23 (0.91) | 1.41 (0.91) | 1.26 (0.88) | 1.34 (0.90) |
| ST weekend | 1.19 (1.05)** | 1.46 (1.15)** | 1.49 (1.08) | 1.61 (1.20) | 1.74 (1.27) | 1.88 (1.13) | 1.90 (1.25) | 2.09 (1.38) |
| ST average per week | 1.03 (0.83)** | 1.29 (0.98)** | 1.23 (0.89) | 1.32 (0.89) | 1.37 (0.93) | 1.52 (0.89) | 1.44 (0.91) | 1.56 (0.95) |

Abbreviations: SD standard deviation, PO playing outside, ST screen time; t-test by sex: * p < 0.05, **p < 0.01
