# Supplementary material for: Effects of screen time and playing outside on anthropometric measures in preschool aged children
Source: PLoS One. 2020 Mar 2;15(3):e0229708. doi: 10.1371/journal.pone.0229708 (PMC7051070; doi:10.1371/journal.pone.0229708)
Supplement: S2 Table — (DOCX) [file pone.0229708.s002.docx]

**S2 Table. Body mass index z-Scores, waist to height ratio at baseline (3 years) and follow-up (6 years) and average time spent playing outside and in front of a screen over the time period, stratified by country.**

|  | Belgium | Spain | Germany | Italy | Poland |
| --- | --- | --- | --- | --- | --- |
| n | 90 | 137 | 61 | 159 | 79 |
| Mean (SD) | | | | | |
| zBMI (baseline) | 0.07 (0.94)* | 0.33 (0.97)* | 0.13 (0.85)* | 0.56 (0.95)* | 0.22 (1.09)* |
| WTH (baseline) | 0.52 (0.03) | 0.53 (0.03) | 0.52 (0.03) | 0.52 (0.03) | 0.52 (0.03) |
| zBMI (follow-up) | -0.05 (0.89)* | 0.47 (1.09)* | -0.11 (0.82)* | 0.52 (1.22)* | 0.28 (1.44)* |
| wth.4 (follow-up) | 0.46 (0.03) | 0.48 (0.03) | 0.46 (0.03) | 0.46 (0.04) | 0.46 (0.04) |
| PO (average) | 2.53 (1.37) | 2.59 (1.22) | 2.57 (1.14) | 2.60 (1.52) | 2.58 (1.70) |
| TV (average) | 1.22 (0.66)* | 1.30 (0.73)* | 0.57 (0.45)* | 1.44 (0.66)* | 1.86 (0.74)* |

Abbreviations: SD standard deviation, zBMI body mass index z-scores according to WHO reference population, WTH waist-to-height ratio PO playing outside, ST screen time; ANOVA by country: * p < 0.001
