# Supplementary material for: Effects of screen time and playing outside on anthropometric measures in preschool aged children
Source: PLoS One. 2020 Mar 2;15(3):e0229708. doi: 10.1371/journal.pone.0229708 (PMC7051070; doi:10.1371/journal.pone.0229708)
Supplement: S3 Table — (DOCX) [file pone.0229708.s003.docx]

**S3 Table. Associations between average time spent playing outside and in front of a screen from 3 to 6 years of age on body mass index z-score and waist-to-height ratio at 6 years; Base model.**

|  | Separate model for playing outside (PO) and screen time (ST) | | | | Mutually adjusted models for PO and ST | |
| --- | --- | --- | --- | --- | --- | --- |
|  | zBMI | WTH | zBMI | WTH | zBMI | WTH |
|  | ß | ß | ß | ß | ß | ß |
|  | 95% CI | 95% CI | 95% CI | 95% CI | 95% CI | 95% CI |
| PO | 0.002 | 0.001 |  |  | 0.001 | 0.001 |
|  | (-0.04, 0.05) | (-0.001, 0.002) |  |  | (-0.04, 0.05) | (-0.001, 0.002) |
|  |  |  |  |  |  |  |
| ST |  |  | 0.20^*^ | 0.01^*^ | 0.19^*^ | 0.01^*^ |
|  |  |  | (0.11, 0.28) | (0.004, 0.01) | (0.11, 0.28) | (0.004, 0.01) |
|  |  |  |  |  |  |  |
| n | 526 | 495 | 526 | 495 | 526 | 495 |
| Adjusted R^2^ | 0.57 | 0.30 | 0.58 | 0.31 | 0.58 | 0.32 |

Note: All ß coefficients from linear regression models; Abbreviations: PO playing outside, ST screen time, 95% CI 95% confidence interval, zBMI BMI z-scores according to WHO reference population, WTH waist-to-height ratio; * p < 0.001
