# Supplementary material for: Effects of screen time and playing outside on anthropometric measures in preschool aged children
Source: PLoS One. 2020 Mar 2;15(3):e0229708. doi: 10.1371/journal.pone.0229708 (PMC7051070; doi:10.1371/journal.pone.0229708)
Supplement: S5 Table — (DOCX) [file pone.0229708.s005.docx]

**S5 Table. Associations between average time spent playing outside and in front of a screen from 3 to 4 years of age on body mass index z-score and waist-to-height ratio at 6 years.**

|  | Separate model for playing outside (PO) and screen time (ST) | | | | Mutually adjusted models for PO and ST | |
| --- | --- | --- | --- | --- | --- | --- |
|  | zBMI | WTH | zBMI | WTH | zBMI | WTH |
|  | ß | ß | ß | ß | ß | ß |
|  | 95% CI | 95% CI | 95% CI | 95% CI | 95% CI | 95% CI |
| PO | -0.03 | -0.002^**^ |  |  | -0.03 | -0.002^*^ |
|  | (-0.08, 0.02) | (-0.004, -0.0001) |  |  | (-0.09, 0.02) | (-0.004, 0.0003) |
|  |  |  |  |  |  |  |
| ST |  |  | 0.13^*^ | 0.005^*^ | 0.14^**^ | 0.01^**^ |
|  |  |  | (0.02, 0.23) | (0.001, 0.01) | (0.04, 0.24) | (0.002, 0.01) |
|  |  |  |  |  |  |  |
| n | 381 | 360 | 369 | 348 | 356 | 335 |
| Adjusted R^2^ | 0.59 | 0.40 | 0.57 | 0.37 | 0.59 | 0.39 |

Note: All ß coefficients from linear regression models, adjusted for country, intervention type, baseline anthropometrics and BMI of mother before birth; WTH additionally adjusted for sex.
Abbreviations: PO playing outside, ST screen time, 95% CI 95% confidence interval, zBMI BMI z-scores according to WHO reference population, WTH waist-to-height ratio; * p < 0.01, **p < 0.001
