# Supplementary material for: Effects of screen time and playing outside on anthropometric measures in preschool aged children
Source: PLoS One. 2020 Mar 2;15(3):e0229708. doi: 10.1371/journal.pone.0229708 (PMC7051070; doi:10.1371/journal.pone.0229708)
Supplement: S6 Table — (DOCX) [file pone.0229708.s006.docx]

**S6 Table Associations between average time spent playing outside and in front of a screen from 3 to 6 years of age on body mass index z-score and waist-to-height ratio at 6 years; Imputed dataset.**

|  | Separate model for playing outside (PO) and screen time (ST) | | | | Mutually adjusted models for PO and ST | |
| --- | --- | --- | --- | --- | --- | --- |
|  | zBMI | WTH | zBMI | WTH | zBMI | WTH |
|  | ß | ß | ß | ß | ß | ß |
|  | 95% CI | 95% CI | 95% CI | 95% CI | 95% CI | 95% CI |
| PO | -0.02 | -0.00 |  |  | -0.02 | -0.00 |
|  | (-0.07, 0.03) | (-0.00, 0.00) |  |  | (-0.07, 0.02) | (-0.00, 0.00) |
|  |  |  |  |  |  |  |
| ST |  |  | 0.17 ** | 0.01 * | 0.18 ** | 0.01 * |
|  |  |  | (0.07, 0.28) | (0.00, 0.01) | (0.07, 0.28) | (0.00, 0.01) |
|  |  |  |  |  |  |  |
| n | 800 | 800 | 800 | 800 | 800 | 800 |
| Adjusted R^2^ | 0.42 | 0.27 | 0.43 | 0.28 | 0.43 | 0.28 |
| m | 5 | 5 | 5 | 5 | 5 | 5 |

Note: All ß coefficients from linear regression models, adjusted for country, intervention type, baseline anthropometrics and BMI of mother before birth.
Abbreviations: PO playing outside, ST screen time, 95% CI 95% confidence interval, zBMI BMI z-scores according to WHO reference population, WTH waist-to-height ratio, m number of imputed datasets
* p < 0.01, **p < 0.001
