# Supplementary material for: Effects of screen time and playing outside on anthropometric measures in preschool aged children
Source: PLoS One. 2020 Mar 2;15(3):e0229708. doi: 10.1371/journal.pone.0229708 (PMC7051070; doi:10.1371/journal.pone.0229708)
Supplement: S7 Table — (DOCX) [file pone.0229708.s007.docx]

**S7 Table. Effects of average time spent playing outside and in front of a screen from 3 to 6 years of age on the odds ratio of having a zBMI >1,zBMI > 2 or WTH > 0.5 at 6 years; Imputed dataset.**

|  | zBMI >1 | zBMI > 2 | WTH > 0.5 |
| --- | --- | --- | --- |
|  | OR 95% CI | OR 95% CI | OR 95% CI |
| PO | 0.86 | 1.01 | 0.91 |
|  | (0.73, 1.02) | (0.79, 1.29) | (0.76, 1.09) |
| ST | 1.61 * | 1.64 | 1.97 ** |
|  | (1.15, 2.24) | (1.00, 2.70) | (1.42, 2.74) |
| n | 800 | 800 | 800 |
| M | 5 | 5 | 5 |

Note: All ß coefficients from linear regression models, adjusted for country, intervention type, baseline anthropometrics and BMI of mother before birth.
Abbreviations: PO playing outside, ST screen time, 95% CI 95% confidence interval, zBMI BMI z-scores according to WHO reference population, WTH waist-to-height ratio, m number of imputed datasets; *p < 0.01, **p < 0.001
