# Supplementary material for: Effects of screen time and playing outside on anthropometric measures in preschool aged children
Source: PLoS One. 2020 Mar 2;15(3):e0229708. doi: 10.1371/journal.pone.0229708 (PMC7051070; doi:10.1371/journal.pone.0229708)
Supplement: S8 Table — (DOCX) [file pone.0229708.s008.docx]

**S8 Table. Effects per hour of average time spent playing outside and
in front of a screen per week from 3 to 6 years of age on the odds of
having a zBMI >1,zBMI > 2 or WTH > 0.5 at 6 years.**

|  | zBMI >1 | zBMI > 2 | WTH > 0.5 |
| --- | --- | --- | --- |
|  | OR (95% CI) | OR (95% CI) | OR (95% CI) |
| PO | **0.98^*^** | 1.01 | 1.00 |
|  | **(0.97, 0.99)** | (1.00, 1.14) | (0.99, 1.01) |
| ST | **1.07^*^** | **1.10^*^** | **1.09^*^** |
|  | **(1.05, 1.10)** | **(1.06, 1.14)** | **(1.06, 1.11)** |
| n | 508 | 508 | 477 |

Note: All odds ratios from logistic regression models, adjusted for
country, intervention type, baseline anthropometrics and maternal
pre-pregnancy BMI (categorized in above and below 25).
* p < 0.001
Abbreviations: PO playing outside, ST screen time,
 95% CI 95% confidence interval, zBMI BMI z-scores according to
WHO reference population, WTH waist-to-height ratio
